# Expanding the Spectrum of *BRAF* Non-V600E Mutations in Thyroid Nodules: Evidence-Based Data from a Tertiary Referral Centre

**DOI:** 10.3390/ijms24044057

**Published:** 2023-02-17

**Authors:** Antonio De Leo, Daniela Serban, Thais Maloberti, Viviana Sanza, Sara Coluccelli, Annalisa Altimari, Elisa Gruppioni, Federico Chiarucci, Angelo Gianluca Corradini, Andrea Repaci, Alessandra Colapinto, Margherita Nannini, Maria A. Pantaleo, Dario de Biase, Giovanni Tallini

**Affiliations:** 1 Solid Tumor Molecular Pathology Laboratory, IRCCS Azienda Ospedaliero-Universitaria di Bologna, 40138 Bologna, Italy; 2 Department of Medical and Surgical Sciences (DIMEC), University of Bologna, 40138 Bologna, Italy; 3 Anatomic Pathology, Department of Medical and Surgical Sciences (DIMEC), University of Bologna, 40138 Bologna, Italy; 4 Pathology Unit, IRCCS Azienda Ospedaliero-Universitaria di Bologna, 40138 Bologna, Italy; 5 Division of Endocrinology and Diabetes Prevention and Care, IRCCS Azienda Ospedaliero-Universitaria di Bologna, 40138 Bologna, Italy; 6 Oncology Unit, IRCCS Azienda Ospedaliero-Universitaria di Bologna, 40138 Bologna, Italy; 7 Department of Pharmacy and Biotechnology, University of Bologna, 40127 Bologna, Italy

**Keywords:** thyroid tumors, *BRAF*, mutation, next-generation sequencing, noncanonical mutation

## Abstract

The BRAF p.V600E mutation represents the most specific marker for papillary thyroid carcinoma and is potentially related to aggressive behavior and persistent disease. *BRAF* alterations other than the p.V600E are less common in thyroid carcinoma and represent an alternative mechanism of BRAF activation with unclear clinical significance. The study aims to describe the frequency and clinicopathologic characteristics of *BRAF* non-V600E mutations in a large cohort (1654 samples) of thyroid lesions characterized by next-generation sequencing. *BRAF* mutations have been found in 20.3% (337/1654) of thyroid nodules, including classic (p.V600E) mutation in 19.2% (317/1654) of samples and non-V600E variants in 1.1% of cases (19/1654). BRAF non-V600E alterations include 5 cases harboring p.K601E, 2 harboring p.V600K substitutions, 2 with a p.K601G variant, and 10 cases with other *BRAF* non-V600E alterations. *BRAF* non-V600E mutations have been reported in one case of follicular adenoma, three cases of conventional papillary thyroid carcinoma, eight cases of follicular variant of papillary carcinomas, one case of columnar cell variant papillary thyroid carcinoma, one case of oncocytic follicular carcinoma, and two bone metastasis of follicular thyroid carcinoma. We confirm that *BRAF* non-V600E mutations are uncommon and typically found in indolent follicular-patterned tumors. Indeed, we show that *BRAF* non-V600E mutations can be found in tumors with metastatic potential. However, in both aggressive cases, the *BRAF* mutations were concomitant with other molecular alterations, such as *TERT* promoter mutation.

## 1. Introduction

Diagnostic thyroidectomy is currently the recommended definitively procedure to exclude malignancy in patients with worrisome thyroid nodules. Molecular testing is commonly used to characterize tumors of follicular cell derivation: the BRAF p.V600E, *RAS* point mutations, and fusion oncogenes (e.g., *RET/PTC* fusion gene) are the most frequent molecular alterations [1,2,3,4,5]. *BRAF* gene codes for a protein belonging to a family of serine-threonine protein kinases that also includes ARAF and CRAF. RAF proteins play an important role in the MAP kinase signaling cascade, one of the pathways involved in cell proliferation and differentiation.

*BRAF* mutations are found in 60–80% of papillary thyroid carcinomas (PTC), and the most frequent alteration is the BRAF p.V600E. This mutation consists of a single nucleotide substitution involving the thymine in position 1799 (transversion, c.1799T > A). This mutation results in a valine to glutamate substitution at amino acid 600 (BRAF p.V600E) that mimics BRAF phosphorylation, causing constitutive activation of the protein. This activation leads to increased and uncontrolled cell proliferation necessary for tumor transformation and promotion. A previous study has demonstrated that *BRAF* mutation may be heterogeneously distributed in PTCs [6]. In PTCs, the BRAF p.V600E mutation has been associated with extrathyroidal extension, lymph nodal and distant metastases, higher stage, and risk of recurrence.

Even if BRAF p.V600E is the most common alteration in PTC, other less common BRAF non-V600E alterations are known, both in codons 599-600-601 or in other residues involved in DFG (Asp-Phe-Gly) motif (Residues 594–596) or activation loop (residues 596–601) [7,8]. Previous studies have demonstrated that mutations involved in codons 599–601 (e.g., p.V600E, p.K601E, and p.V600K) or codon 469 (e.g., p.G469V) may significantly influence BRAF activity [9,10,11], causing constitutive activation of the protein. On the contrary, substitutions present in other codons of *BRAF* genes do not seem to have the oncogenic potential [7,9]. According to their activity, *BRAF* mutations have been classified into three functional classes [9,12,13]: (i) class 1 *BRAF* mutations (BRAF p.V600), RAS independent, signal as monomers, and characterized by strong activity of BRAF kinase domain; (ii) class 2 *BRAF* alterations, RAS independent, signal as constitutive dimers, and with intermediate to high activity of BRAF kinase domain; (iii) class 3 *BRAF* alterations, RAS dependent, characterized by low or absent kinase activity.

The aim of the present study was to report the experience of the Bologna molecular pathology laboratory regarding the frequency of *BRAF* non-V600E alterations in a large clinical cohort of thyroid nodules.

## 2. Results

A total of 336 of the 1654 thyroid samples analyzed (20.3%) harbored a *BRAF* mutation (Table 1). Of 336 mutations, 317 (94.3%) were the canonical BRAF p.V600E variant, and the remaining 19 (5.7%) were *BRAF* non-V600E mutations (Table 1). One of the 19 non-V600E mutations was on exon 11, and the remnants were on exon 15. (Table 2).

The *BRAF* mutational status and clinicopathological features of the specimens harboring non-V600E mutation are reported in Table 2 and Table 3.

A total of 20 mutations were detected in 19 cases. In one case (case#1—Table 2), a BRAF p.V600E mutation was coexistent with a *BRAF* non-V600E variant (p.K601_W604del—Table 2). Of the 19 non-V600E mutations, 11 (57.9%) were single nucleotide variants (SNVs), and 8 (42.1%) were “indel” variants (Table 2). Nine of the non-V600E mutations were p.K601E (5 cases), p.V600K (2 cases), or p.K601G (2 cases), while the remaining 10 variants were all different between them (Table 2 and Table 3).

**Figure 1:**
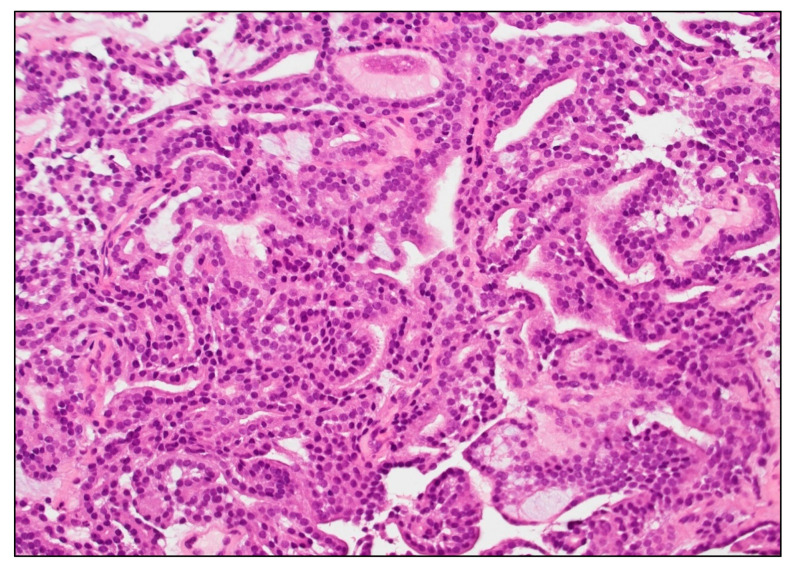
Follicular thyroid carcinoma metastatic to bone (sacrum), case 3 Table 1, harboring BRAF p.K601N. The tumor is follicular patterned, well-differentiated, and lacks the nuclear atypia of papillary carcinoma, mitotic activity, and tumor necrosis. Magnification: 100×.

Follicular variant papillary thyroid carcinoma (FV-PTC) was the most common histological subtype harboring *BRAF* non-V600E mutations (8 of 19 cases, 42.1%). *BRAF* non-V600E mutations were also observed in one case of follicular adenoma (5.3%), three cases of conventional papillary thyroid carcinoma (15.8%), one case of columnar cell variant papillary thyroid carcinoma (5.3%), one case of oncocytic carcinoma (5.3%), and two bone metastases of follicular thyroid carcinoma (10.5%). Tumor capsule was present in five cases (Table 3 and Table 4). An extrathyroidal extension was observed in one classic papillary carcinoma (CL-PTC) (case #19—Table 4), while vascular invasion was detectable in three cases (Table 3 and Table 4). Cases with *BRAF* non-V600E mutations were mostly pT1 (10 of 13, 76.9%), including seven pT1a and three pT1b (Table 3). According to the AJCC (American Joint Committee on Cancer) prognostic stage, eleven cases were stage I, and one was stage II (Table 3 and Table 4). The vast majority of the samples harboring non-V600E were indolent thyroid lesions, but in two cases, distant (bone) metastases were detected at presentation (case #2, #3—Table 2 and Table 4, Figure 1).

## 3. Discussion

*BRAF* encodes the BRAF protein, which belongs to a family of serine-threonine protein kinases, including ARAF and CRAF. RAF proteins play an important role in the MAP kinase signaling cascade, one of the main pathways that regulate cell proliferation and differentiation. Mutant *BRAF* has been implicated in the pathogenesis of different types of cancer, including thyroid carcinoma, colorectal carcinoma, non-small cell lung carcinoma, ovarian carcinoma, gliomas, melanoma, and gastrointestinal stromal tumor (GIST) [14].

In this study, we investigated the prevalence and clinical significance of *BRAF* non-V600E variants in a large cohort of cases from a tertiary referral center. Next-generation sequencing allowed the detection of *BRAF* non-V600E mutations in 1.2% of 1654 thyroid nodules. *BRAF* non-V600E mutations are uncommon. They are typically found in indolent follicular-patterned tumors, and thus—in spite of being *BRAF* mutated forms—belong to the spectrum of thyroid tumors with *RAS*-like features (instead of belonging to the spectrum of *BRAF*-V600E-like tumors). In the TCGA cohort, of 240 mutated cases, 5 (2.1%) harbored non-V600E mutations. All of them were FV-PTC with p.K601E substitution or inframe deletions/insertion (cBioPortal—“Thyroid Carcinoma—TCGA”, Firehose Legacy).

BRAF p.K601E mutation represented the most common BRAF non-V600E alteration and, in agreement with other published case series, was only detected in follicular-patterned tumors (see Table 4) [15,16]. BRAF p.K601E has been reported in a few cases of follicular thyroid carcinoma [11,17,18], as well as in one case of NIFTP [19] and four cases of follicular adenoma [11,20,21,22]. Similar to previous reports, we found BRAF p.K601E in follicular variant PTC (four cases) as well as in one case of follicular adenoma. In our series, K601E-mutant FV-PTC showed no aggressive features, such as extrathyroidal extension, vascular invasion, or metastases, supporting the notion that BRAF p.K601E mutated thyroid nodules are typically indolent, in contrast to BRAF p.V600E mutated carcinomas [23,24]. In addition to BRAF p.K601E, we identified other non-V600E mutations not only in follicular-patterned tumors (two FV-PTC and three FTC) but also in three classical PTCs and in one CCV-PTC. Interestingly, a recent study reported that CCV-PTC appears to harbor a higher incidence of non-V600E variants [25].

Among *BRAF* non-V600E and non-K601E mutations, BRAF p.T599del was detected in one FV-PTC. This mutation has previously been reported in an adenomatous goiter [18,26]. BRAF p.K601_W604del was found in one of our FV-PTC cases. The mutation has previously been reported in papillary thyroid carcinoma [26,27]. *BRAF* p.T599_V600insEAT, detected in one classical PTC from our cohort, has never been previously reported in thyroid lesions but was identified in a single case of Langerhans cell histiocytosis [26,28]. We found one *BRAF* exon 11 mutation (p.G469S) in a case of FV-PTC. This mutation, too, had never been previously reported in thyroid lesions, but it was identified in hematopoietic and in solid tumors (e.g., melanomas, lung adenocarcinoma, and bile duct adenocarcinoma) [26].

The vast majority of the samples harboring *BRAF* non-V600E mutations were indolent thyroid lesions. However, we identified *BRAF* non-V600E mutations in two FTC patients with distant (bone) metastases at presentation. One FTC harbored *BRAF* p.A598_T599insILA and the other *BRAF* p.K601N. *BRAF* p.A598_T599insILA has never been previously reported. *BRAF* p.K601N has been reported in rare cases of FTC, thyroid lymphoma, lung carcinoma, colorectal carcinomas, and melanoma [26,29,30]. Intriguingly, the two cases with aggressive behavior harbored another mutation other than *BRAF* non-V600E: in one case, a pathogenic *TERT* promoter mutation was detected (case #2, Table 2); the other case harbored a 19q imbalance (case #3, Table 2) [30].

In conclusion, our results expand the spectrum of *BRAF* non-V600E mutations in thyroid nodules. We have confirmed that *BRAF* non-V600E mutations are uncommon and typically found in indolent follicular-patterned tumors. We report for the first time three *BRAF* non-V600E variants previously undetected in thyroid tumors, including one concomitant with *TERT* mutation and associated with distant metastasis. Indeed, we show that *BRAF* non-V600E mutations can be found in tumors with metastatic potential if associated with other molecular alterations. Thus, *BRAF* non-V600E mutated tumors should be thoroughly investigated, looking for thyroid tumor progression-related molecular changes such as *TERT* promoter mutation.

## 4. Materials and Methods

### 4.1. Case Selection

Data about 1654 thyroid nodules subjected to *BRAF* mutation analysis from January 2011 until June 2019 were collected from the database of the solid tumor molecular athology laboratory (Bologna, Italy). Analyses were performed starting from fine-needle aspiration specimens (“Direct fine needle aspiration cytology specimen” or “Stained cytological smear”) or surgical material (formalin-fixed and paraffin-embedded tissue—FFPE). The cytopathological and histopathological review was performed by two expert pathologists (GT, ADL) according to World Health Organization classification of thyroid malignancy [31].

### 4.2. DNA Extraction and BRAF Analysis

DNA from FFPE specimens was extracted starting from 2 to 4 serial 10 µm-thick sections using the QuickExtract FFPE DNA extraction kit (LGC Biosearch Technologies, Berlin, Germany), scraping the area of interest, according to the selection performed by a pathologist on final hematoxylin and eosin (E&H) slide. DNA was quantified using Qubit dsDNA BR assay kit (Thermo Fisher Scientific, Waltham, MA, USA). DNA from cytological specimens was extracted using the MasterPure DNA purification kit (LGC Biosearch Technologies, Berlin, Germany), according to the manufacturer’s instructions. Sequencing was performed using different types of NGS: 454 GS-Junior (Roche Diagnostics, Basel, Switzerland) [6], or multigene custom panel by MiSeq (Illumina Inc., San Diego, CA, USA) and Gene Studio S5 sequencers (Thermo Fisher Scientific, Waltham, MA, USA) [32]. Results were analyzed using the Amplicon variant analyzer tool (Roche, Basel, Switzerland), Illumina Variant Studio (Illumina Inc., San Diego, CA, USA), IonReporter tool (ThermoFisher Scientific, Waltham, MA, USA), and the Integrative Genomics Viewer v.2.3 (IGV) tool http://software.broadinstitute.org/software/igv/, accessed on 30 November 2022). ACMG classification for the mutations was retrieved using the Varsome Database (https://varsome.com/, accessed on 30 November 2022).

## Figures and Tables

**Table 1:** *BRAF* results in the cohort analyzed.

BRAF-Mutation	Number of Cases	Frequency (%)
p.V600E	317	19.2
Non-V600E	19	1.1
WT	1230	74.4
NA	88	5.3
TOTAL	1654	100

WT, Wild-Type; NA, not assessable due to low quality/quantity DNA.

**Table 2:** Cases harboring BRAF non-V600E Mutations.

#	Age	Sex	Site	Starting Material	Cytopathologycal Report	Histopathological Report	BRAF-Mutation	ACMG Classification	BRAF Class	Other Alterations
1	64	M	Thy	Cytological smear + FFPE	Thy3f	FV-PTC	p.V600E p.K601_W604del	P NA	1 NA	/
2	73	M	Bone	FFPE	NA	FTC (metastasis)	p.A598_T599insILA	NA	NA	*TERT* c.-124C > T
3	85	F	Bone	FFPE	NA	FTC (metastasis)	p.K601N	P	2	Chr19 imbalance
4	38	M	Thy	FFPE	Thy3f	FV-PTC	p.K601E	P	2	/
5	56	F	Thy	Cytological smear	Thy3a	FV-PTC	p.K601E	P	2	/
6	33	F	Thy	Direct FNA	Thy4	CL-PTC	p.V600_S605delinsDT	NA	NA	/
7	68	M	Thy	Cytological smear	Thy3a	FV-PTC	p.T599del	NA	NA	/
8	77	F	Thy	Direct FNA	Thy3a	FV-PTC	p.V600_K601insNTV	NA	NA	
9	36	F	Thy	Direct FNA	Thy3f	FA	p.K601E	P	2	/
10	66	F	Thy	FFPE + Direct FNA	Thy3a	FV-PTC	p.K601E	P	2	/
11	58	F	Thy	Direct FNA	Thy3a	NA *	p.K601G	P	2	/
12	60	M	Thy	Direct FNA	Thy3a	NA *	p.K601G	P	2	/
13	40	M	Thy	Cytological smear	Thy3f	FV-PTC	p.K601E	P	2	/
14	53	F	Thy	Cytological smear	Thy3a	NA *	p.V600delinsNM	NA	NA	/
15	70	F	Thy	Direct FNA	Thy3a	OC	p.V600K	P	1	/
16	69	F	Thy	Direct FNA	Thy4	CL-PTC	p.V600K	P	1	/
17	55	F	Thy	FFPE	NA	CCV-PTC	p.V600_R603delfs	NA	NA	/
18	43	F	Thy	Direct FNA	Thy3a	CL-PTC	p.T599_V600insEAT	NA	NA	/
19	58	F	Thy	Direct FNA	Thy3a	FV-PTC	p.G469S	P	NA	/

FFPE: Formalin-fixed and Paraffin-Embedded; FNA: Fine Needle Aspiration; Chr: chromosome; Thy: Thyroid; NA: Not Available; * Loss at follow-up; P: Pathogenic; PTC: Papillary Thyroid Carcinoma; CL: Classical; FV: Follicular Variant; CCV: Columnar Cell Variant FA: Follicular Adenoma; FTC: Follicular Thyroid Carcinoma; OC: Oncocytic Carcinoma; Note: FV-PTC includes invasive and noninvasive tumors (i.e., NIFTP: Noninvasive follicular thyroid neoplasm with papillary like nuclear features).

**Table 3:** Summary of clinicopathological characteristics of the 19 cases harboring BRAF non-V600E alteration.

Clinicopathologic Characteristics	n (%)
Age	
median (range)	59.6 (33–80)
Sex (n = 19)	
Female	14 (73.7%)
Male	5 (26.3%)
Size (mm)	
median (range)	14 (1–80)
Non-V600E *BRAF* Alterations (n = 19)	
p.K601E	5 (26%)
p.V600K	2 (10.5%)
p.K601G	2 (10.5%)
Other *BRAF* non-V600E	10 (53%)
Histology (n = 19)	
Follicular adenoma	1 (5.3%)
CL-PTC	3 (15.8%)
FV-PTC	8 (42.1%)
CCV-PTC	1 (5.3%)
FTC (metastasis)	2 (10.5%)
OC	1 (5.3%)
NA	3 (15.8%)
Tumor capsule (n= 13)	
Yes	5 (38.4%)
No	8 (61.5%)
Extrathyroidal extension (n= 13)	
Yes	1 (7.7%)
No	12 (92.3%)
Vascular invasion (n= 13)	
Yes	3 (23.1%)
No	10 (76.9%)
pT (n= 13)	
pT1a	6 (46.1%)
pT1b	4 (30.8%)
pT2	2 (15.4%)
pT3	1 (7.7%)
AJCC stages (n= 15)	
I	12 (80%)
II	1 (6.6%)
IVB	2 (13.3%)
Metastasis (n = 15)	
Present	2 (13.3%)
Absent	13 (86.7%)

NA: Not Available; PTC: Papillary Thyroid Carcinoma; CL: Classical; FV: Follicular Variant; CCV: Columnar Cell Variant FA: Follicular Adenoma; FTC: Follicular Thyroid Carcinoma; OC: Oncocytic Carcinoma; Note: FV-PTC includes invasive and noninvasive tumors (i.e., NIFTP: Noninvasive follicular thyroid neoplasm with papillary like nuclear features).

**Table 4:** Description of the clinicopathological characteristics of the 19 cases harboring BRAF non-V600E alterations.

Case	Histological Type	Size (mm)	Stage	Vascular Invasion	Tumor Capsule	Extrathyroidal Extension	Necrosis	AJCC Prognostic Stage	*BRAF*
1	FV-PTC	23	II	Yes	Yes	No	No	I	p.V600E p.K601_W604del
2	FTC (metastasis)	NA	NA	NA	NA	NA	NA	IVB	p.A598_T599insILA
3	FTC (metastasis)	NA	NA	NA	NA	NA	NA	IVB	p.K601N
4	FV-PTC	9	Ia	No	No	No	No	I	p.K601E
5	FV-PTC	2	Ia	No	No	No	No	I	p.K601E
6	CL-PTC	10	Ia	No	No	No	No	I	p.V600_S605delinsDT
7	FV-PTC	15	Ib	NA	NA	NA	NA	I	p.T599del
8	FV-PTC	9	Ia	No	No	No	No	I	p.V600_K601insTV
9	FA	35	NA	NA	NA	NA	NA	NA	p.K601E
10	FV-PTC	10	Ia	No	No	No	No	I	p.K601E
11	NA *	9.6	NA	NA	NA	NA	NA	NA	p.K601G
12	NA *	15	NA	NA	NA	NA	NA	NA	p.K601G
13	FV-PTC	4	Ia	No	No	No	No	I	p.K601E
14	NA *	13	NA	NA	NA	NA	NA	NA	p.V600delinsNM
15	OC	16	Ib	No	Yes	No	No	I	p.V600K
16	CL-PTC	15	Ib	No	No	No	No	I	p.V600K
17	CCV-PTC	45	III	Yes	Yes	No	Yes	II	p.V600fs
18	CL-PTC	16	Ib	Yes	Yes	Yes	No	I	p.T599_V600insEIAT
19	FV-PTC	32	II	No	Yes	No	No	I	p.G469S

NA: Not Available; * Loss at follow-up; PTC: Papillary Thyroid Carcinoma; CL: Classical; FV: Follicular Variant; CCV: Columnar Cell Variant FA: Follicular Adenoma; FTC: Follicular Thyroid Carcinoma; OC: Oncocytic Carcinoma; Note: FV-PTC includes invasive and noninvasive tumors (i.e., NIFTP: Noninvasive follicular thyroid neoplasm with papillary like nuclear features).

## Data Availability

All data is contained within the article.
